# The reliability of weight‐for‐length/height Z scores in children

**DOI:** 10.1111/mcn.12124

**Published:** 2014-05-01

**Authors:** Martha K. Mwangome, James A. Berkley

**Affiliations:** ^1^ KEMRI/Wellcome Trust Research Programme Kilifi Kenya; ^2^ Centre for Clinical Vaccinology & Tropical Medicine University of Oxford Oxford UK

**Keywords:** reliability, weight‐for‐length, weight‐for‐height, children

## Abstract

The World Health Organisation (WHO) recommends weight‐for‐length/height (WFL/H), represented as a Z score for diagnosing acute malnutrition among children aged 0 to 60 months. Under controlled conditions, weight, height and length measurements have high degree of reliability. However, the reliability when combined into a WFL/H Z score, in all settings is unclear. We conducted a systematic review of published studies assessing the reliability of WFL/Hz on PubMed and Google scholar. Studies were included if they presented reliability scores for the derived index of WFL/Hz, for children under 5 years. Meta‐analysis was conducted for a pooled estimate of reliability overall, and for children above and below 24 months old. Twenty six studies on reliability of anthropometry were identified but only three, all community‐based studies, reported reliability scores for WFL/Hz. The overall pooled intra‐class correlation coefficient (ICC) estimate for WFL/Hz among children aged 0 to 60 months was 0.81 (95% CI 0.64 to 0.99). Among children aged less than 24 months the pooled ICC estimate from two studies was 0.72 (95% CI 0.67 to 0.77) while the estimate reported for children above 24 months from one study was 0.97 (95% CI 0.97 to 0.99). Although WFL/Hz is recommended for diagnosis of acute under nutrition among children below 5 years, information on its reliability in all settings is sparse. In community settings, reliability of WFL/Hz is considerably lower than for absolute measures of weight and length/height, especially in younger children. The reliability of WFL/Hz needs further evaluation.

## Introduction

Childhood undernutrition is responsible for approximately 3.1 million child deaths each year (Black *et al*. 2013) and between 11 and 41% of hospital admissions (Bejon *et al*. 2008). The global prevalence of severe acute undernutrition (SAM) has been estimated at 19 million children (Black *et al*. 2008; Kerac *et al*. 2011). For the assessment of acute undernutrition amongst children ages 0 to 60 months, the WHO recommends the use of weight‐for‐length/height (WFL/H) represented as a Z score or a percentile. In children aged 6 to 60 months unadjusted MUAC is also recommended (WHO & UNICEF 2009). Weight and length/height are measured separately before being converted to WFL/Hz by examining look‐up tables or using computer software. It is possible that the process of converting weight and length/height into WFL/Hz results in decreased reliability for WFL/Hz compared to the individual measures because during the conversion, errors from both measures are incorporated. In anthropometric assessment, reliability is defined as the consistency of results when repeated examinations are performed by the same (intra‐observer) or different (inter‐observer) observers under the same conditions (Hennekens & Buring 1987). Reliability of WFLz is an important requirement as it directly impacts on the admission and discharge criterion for children in nutrition interventions.

Previous studies of reliability have been conducted within a carefully controlled hospital or research environment using highly qualified and carefully trained health workers, and have reported high reliability scores for absolute measures of weight, length/height and MUAC (WHO 2006, Johnson *et al*. 2009). However the reliability of derived indices such as WFL/Hz and HFAz has generally not been reported. In resource poor countries, community health workers (CHWs) are used to deliver community health services including anthropometric assessment, basic nutrition counseling and education at household level (Ministry of Health 2006). We set out to review published literature on reliability of WFL/H performed by community health workers and where WFL/H Z score conversion was performed using computer software to eliminate look‐up errors.

### Key messages

- For the assessment of acute undernutrition amongst children ages 0 to 60 months, the use of weight‐for‐length/height (WFL/H) represented as a Z score or a percentile is recommended.
- There are more published articles reporting the inter‐observer reliability estimates for absolute weight and height/length measures than reliability estimates for WFL/Hz.
- All studies reported high reliability scores for absolute measures of weight and length/height but this did not translate to high scores for the combined index of WFL/Hz.
- Future studies on reliability of anthropometric measures to present data on derived indicators of undernutrition to validate their usability across different settings.

## Objective

To evaluate the inter‐observer variation of the WHO recommended anthropometric criteria for assessing acute undernutrition (weight‐for‐length/height) in children aged 0 to 60 months.

## Methods

### Search strategy

The search was conducted on PubMed using the following search terms: ‘use OR reliability OR repeatability OR “inter‐observer” OR “inter‐observer” OR “inter observer”) and (anthropom* OR “weight for height” OR “weight for length”) and (infants OR children)’. From the search, relevant titles and abstracts were identified while those obviously irrelevant were excluded. The remaining articles were reviewed to identify those reporting reliability scores for derived indicators of wasting in children. Additional studies not picked up by the search strategy were identified from reviewing the list of references from the full texts.

### Inclusion/exclusion criteria

Reliability studies were considered in this review if they reported a reliability estimate for WFL/H using either the technical error of measurement (TEM^1^ ) and/or the intra‐class correlation coefficient (ICC^2^ ). Pooled ICC was calculated by meta‐analysis using a random effect model assuming heterogeneity between sites measuring different populations (Shrout & Fleiss 1979).

## Results

**Figure 1:**
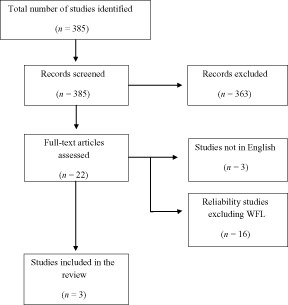
Flow diagram showing identification of studies.

Of the 390 articles identified in the initial search, 23 reported the reliability of weight and or height/length among children. Three additional studies were identified from reviewing the reference list of the identified articles (Warner 2000; Morris & Flores 2002; Jamaiyah *et al*. 2010). Of these 26 publications, 21 reported reliability scores for absolute anthropometric measures such as weight, height/length, mid‐upper arm circumference and head circumference but not for their composite indices like WFA or WFL/H (Engstrom 1988; Bhushan & Paneth 1991; Voss *et al*. 1991; Rosenberg *et al*. 1992; Voss & Bailey 1994; Doull *et al*. 1995; Johnson *et al*. 1997; Johnson *et al*. 1998; Poustie *et al*. 2000; Warner 2000; Bradley *et al*. 2001; Morris & Flores 2002; Vegelin *et al*. 2003; Wang *et al*. 2003; WHO 2006; Frainer *et al*. 2007; Johnson *et al*. 2009; Jamaiyah *et al*. 2010; Ngirabega *et al*. 2010; Stomfai *et al*. 2011; West *et al*. 2011). One study reported reliability scores for WFA (Lima *et al*. 2010) and another reported the body‐mass index (BMI) (Oza‐Frank *et al*. 2012) but not for WFL/Hz. Three studies (Velzeboer *et al*. 1983; Ayele *et al*. 2012b; Mwangome *et al*. 2012) reported the reliability estimates for WFL/H thus meeting the inclusion criteria and were included in the review (Fig. 1). In the Velzeboer study, (Velzeboer *et al*. 1983), since the confidence intervals (CI) for the ICC estimates were not provided in the published article, they were recalculated using the formulae provided on page 158 of her thesis (Velzeboer 1979). In all the studies, community members (farmers, community health workers or health promoters) with minimal knowledge of anthropometry were trained for not less than 2 days on how to measure weight, length/height and MUAC among children. No formal standardization tests were done instead a practical session was organized where the trainer performed an assessment of measuring techniques by observing and correcting skill and comparing their measures to trainees measures. Thereafter, each measurer was instructed to take repeated measures of children individually. In all the studies, the ICC for the composite measure of WFL/Hz is considerably lower than that of the single measures of weight and length/height (Table 1).

**Table 1:** A summary of inter‐observer reliability data from three community‐based studies

Author, year	Location	Age range	(No)	Index	TEM	ICC	Observer
Mwangome *et al*. 2012	Kenya	0 to 6 months	924	Weight	–	0.98 (0.98 to 0.99)	Community health workers
				Length	–	0.95 (0.95 to 0.96)	
				WFLz	–	**0.71 (0.68 to 0.74)**	
Ayele *et al*. 2012a, 2012b	Ethiopia	0 to 24 months	28	Weight	1.28 Kg	0.99 (0.99 to 1.0)	Community drawn anthropometrist
				Length	1.04 cm	0.99 (0.99 to 1.0)	
				WFLz	0.4	**0.79 (0.66 to 0.94)**	
		24 to 60 months	61	Weight	0.08 Kg	0.99 (0.99 to 1.0)	
				Height	0.38 cm	0.99 (0.99 to 1.0)	
				WFHz	0.1	**0.97 (0.97 to 0.99)**	
Velzeboer *et al*. 1983	Guatemala	12 to 60 months	162	WFL/Hz	–	**0.78 (0.72 to 0.84)**	Minimally trained health workers

IQR, inter quartile range; TEM, technical error of measurement; ICC, intra‐class correlation coefficient; WFLz, weight‐for‐length Z scores; WFHz, weight‐for‐height Z scores.

**Figure 2:**
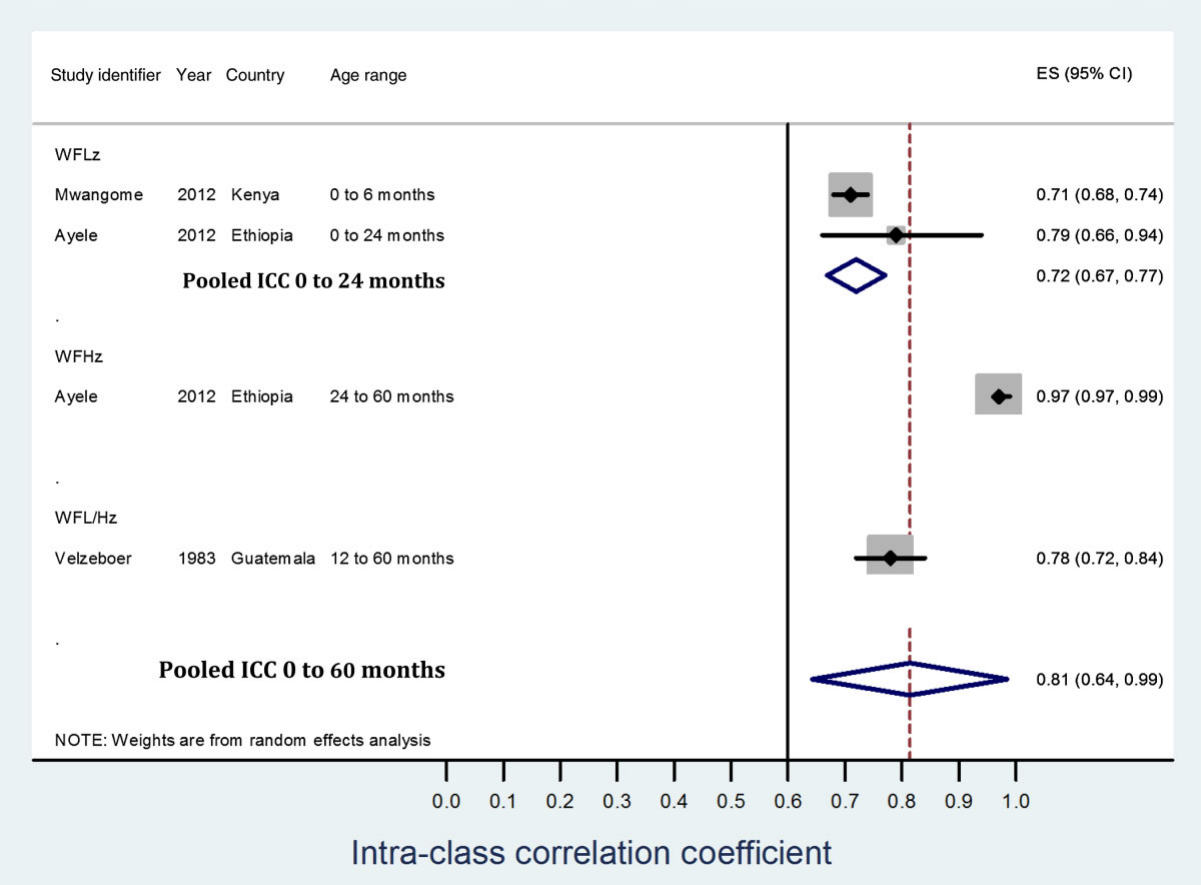
Pooled intra‐class correlation coefficient for WFL/Hz among children below 5 years.

In Ayele's study (Ayele *et al*. 2012b), age‐specific reliability scores (above and below 24 months) were made available (Ayele *et al*. 2012a) and thus the WFL/Hz pooled ICC estimate for children below 24 months was estimated at 0.72 (95% CI 0.67 to 0.77) while for children below 5 years was 0.81 (95% CI 0.64 to 0.99) (Fig. 2). Only one study (Ayele *et al*. 2012b)presented reliability scores for WFL/Hz estimated by TEM (Table 1).

## Discussion

There are no published data on reliability of WFL/Hz as evaluated in strictly controlled conditions. The studies identified in this review assessed the reliability of WFL/Hz within a community setting. We found more articles reporting the inter‐observer reliability estimates for absolute measures than reliability estimates for indicators of undernutrition. The limited data may explain the wide confidence interval observed for the pooled ICC estimate for all the studies included in this review (Fig. 1). More data on the reliability of indicators of undernutrition among children needs to be generated to facilitate our understanding on their usability.

Previously, it was assumed that acceptable intra and inter‐observer reliability estimates for weight and length/height could be translated to acceptable reliable scores for WFL/Hz measure. Data from this review is not supportive of this assumption. In all the three studies, high reliability scores were reported separately for absolute measures of weight and length/height but not for the combined index of WFL/Hz. It is possible that WFL/Hz is sensitive to individual variations in the component measures of weight and length (Mwangome *et al*. 2012) and that these individual variations are compounded when weight and length are presented as a ratio resulting in a lower reliability score for WFL/H (Velzeboer *et al*. 1983). This hypothesis would need to be validated using data from more controlled conditions.

The reliability scores for WFLz in younger children (0 to 24 months) appear to be lower than that of WFHz among older children (24 to 59 months); the confidence interval for pooled ICC on younger children; <24 months, and the ICC for older children; >24 months, did not overlap. This observation indicates increased levels of measurement error among the younger children likely because they cannot co‐operate with measurements as well as the older children (Walker *et al*. 2013). Additionally, for infants aged below 6 months, the infantile position may hinder the reliable measuring of length. Studies evaluating the reliability of anthropometry among children within a community settings have indicated higher likelihood of variation in measuring length compared to weight (Velzeboer *et al*. 1983; Mwangome *et al*. 2012) and have pointed to the complexity in technique, equipment and the nature of the infant as possible sources of error (Velzeboer *et al*. 1983; Walker *et al*. 2013). It may be that ensuring reliable measures of length/height through introducing more intensive training of health workers, using paired observer to compare readings (WHO 2006) using electronics for direct data entry and using simplified look‐up Z score charts (Kerac *et al*. 2009) will increase the reliability of WFL/Hz. Notwithstanding; WFL/H measure has additional characteristics that make it unattractive to minimally trained health workers in poorer settings; it's equipment are more costly to buy, install and maintain, it consumes a lot of time to measure and interpret as weight and length/height are measured separately and health workers are required to looked up a table to interpret (Myatt *et al*. 2006). Thus in addition to finding reliable measure of length/height in children, research should focus on identifying a simpler and possibly a more reliable assessment tool in place of WFL/Hz. More data is needed to affirm these observations.

Although high reliability of WFL/Hz does not necessarily ensure validity, low reliability means that there is an increased chance of underestimating or failing to detect undernutrition among children using this tool. This observation is of important concern to public health practitioners and policy makers.

## Conclusion

Although WFL/Hz is recommended as the anthropometric criteria for diagnosis of acute undernutrition among children below 5 years, there are hardly any data to describe its reliability in either controlled or practical settings. Future studies on reliability of anthropometric measures present data on derived indicators of undernutrition such as WFL/Hz, WFAz and HFAz, in addition to that of absolute measures. This will inform, clarify and validate their usability across different settings. In the meantime, training and application on the use of more reliable anthropometric indicators of acute undernutrition in endemic settings should be encouraged as the poor reliability of WFL/Hz amongst infants under field conditions may limit its interpretation in this age group.

## Source of funding

This work was supported by Kenya Medical Research Institute (KEMRI) through a strategic award (084538) and personal fellowship (083576) from the Wellcome Trust and is published with the permission of the Director of KEMRI.

## Conflict of interest

The authors declare that they have no conflicts of interest.

## Contributions

The study was conceived, designed and executed by MM under the supervision of JB. Both authors were involved in data acquisition, analysis, interpretation and manuscript writing. They have read and approved the final manuscript.

## Footnotes

1 The smaller the TEM the more reliable the measure.

2 ICC is a number between 0 and 1 and the closest the estimate is to 1 the better the reliability.

## References

[mcn12124-bib-0001] Ayele B. , Aemere A. , Gebre T. , Tadesse Z. , Stoller N.E. , See C.W. *et al* (2012a) *Reliability of measurements performed by community‐drawn anthropometrists from rural Ethiopia* . [Online]. Available at: http://www.plosone.org/annotation/listThread.action?root=50461 (Accessed 1 July 2013). 10.1371/journal.pone.0030345 PMC3265464 22291939

[mcn12124-bib-0002] Ayele B. , Aemere A. , Gebre T. , Tadesse Z. , Stoller N.E. , See C.W. *et al* (2012b) Reliability of measurements performed by community‐drawn anthropometrists from rural Ethiopia. PLoS ONE 7, e30345 [Online]. Available at: http://www.ncbi.nlm.nih.gov/pubmed/22291939 (Accessed 30 July 2012). 22291939 10.1371/journal.pone.0030345 PMC3265464

[mcn12124-bib-0003] Bejon P. , Mohammed S. , Mwangi I. , Atkinson S.H. , Osier F. , Peshu N. *et al* (2008) Fraction of all hospital admissions and deaths attributable to malnutrition among children in rural Kenya. The American Journal of Clinical Nutrition 88, 1626–1631. 19064524 10.3945/ajcn.2008.26510 PMC2635111

[mcn12124-bib-0004] Bhushan V. & Paneth N. (1991) The reliability of neonatal head circumference measurement. Journal of Clinical Epidemiology 44, 1027–1035. 1940995 10.1016/0895-4356(91)90004-s

[mcn12124-bib-0005] Black R.E. , Allen L.H. , Bhutta Z.A. , Caulfield L.E. , De Onis M. , Ezzati M. *et al* (2008) Maternal and child undernutrition: global and regional exposures and health consequences. Lancet 371, 243–260. 18207566 10.1016/S0140-6736(07)61690-0

[mcn12124-bib-0006] Black R.E. , Victora C.G. , Walker S.P. , Bhutta Z.A. , Christian P. , De Onis M. *et al* (2013) Maternal and child undernutrition and overweight in low‐income and middle‐income countries. Lancet 382, 427–451. 23746772 10.1016/S0140-6736(13)60937-X

[mcn12124-bib-0007] Bradley J.L. , Brown J.E. & Himes J.H. (2001) Reliability and validity of parental measurements of infant size. American Journal of Human Biology 13, 275–279. 11460874 10.1002/1520-6300(200102/03)13:2<275::AID-AJHB1039>3.0.CO;2-3

[mcn12124-bib-0008] Doull I.J. , McCaughey E.S. , Bailey B.J. & Betts P.R. (1995) Reliability of infant length measurement. Archives of Disease in Childhood 72, 520–521. 7618938 10.1136/adc.72.6.520 PMC1511143

[mcn12124-bib-0009] Engstrom J.L. (1988) Assessment of the reliability of physical measures. Research in Nursing and Health 11, 383–389. 3231740 10.1002/nur.4770110606

[mcn12124-bib-0010] Frainer D.E. , Adami F. , Vasconcelos Fde A. , Assis M.A. , Calvo M.C. & Kerpel R. (2007) [Standardization and reliability of anthropometric measurements for population surveys]. Archivos Latinoamericanos de Nutricion 57, 335–342. 18524317

[mcn12124-bib-0011] Hennekens C.H. & Buring J.E. (1987) Epidemiology in Medicine. Lippincott Williams & Wilkins: Boston, MA.

[mcn12124-bib-0012] Jamaiyah H. , Geeta A. , Safiza M.N. , Khor G.L. , Wong N.F. , Kee C.C. *et al* (2010) Reliability, technical error of measurements and validity of length and weight measurements for children under two years old in Malaysia. The Medical Journal of Malaysia 65 (Suppl. A), 131–137. 21488474

[mcn12124-bib-0013] Johnson T.S. , Engstrom J.L. & Gelhar D.K. (1997) Intra‐ and interexaminer reliability of anthropometric measurements of term infants. Journal of Pediatric Gastroenterology and Nutrition 24, 497–505. 9161941 10.1097/00005176-199705000-00001

[mcn12124-bib-0014] Johnson T.S. , Engstrom J.L. , Warda J.A. , Kabat M. & Peters B. (1998) Reliability of length measurements in full‐term neonates. Journal of Obstetric, Gynecologic, and Neonatal Nursing 27, 270–276. 10.1111/j.1552-6909.1998.tb02649.x 9620819

[mcn12124-bib-0015] Johnson W. , Cameron N. , Dickson P. , Emsley S. , Raynor P. , Seymour C. *et al* (2009) The reliability of routine anthropometric data collected by health workers: a cross‐sectional study. International Journal of Nursing Studies 46, 310–316. 19019368 10.1016/j.ijnurstu.2008.10.003

[mcn12124-bib-0016] Kerac M. , Seal A. , Blencowe H. & Bunn J. (2009) Improved assessment of child nutritional status using target weights and a novel, low‐cost, weight‐for‐height slide chart. Tropical Doctor 39, 23–26. 19211417 10.1258/td.2008.080096

[mcn12124-bib-0017] Kerac M. , Blencowe H. , Grijalva‐Eternod C. , McGrath M. , Shoham J. , Cole T.J. *et al* (2011) Prevalence of wasting among under 6‐month‐old infants in developing countries and implications of new case definitions using WHO growth standards: a secondary data analysis. Archives of Disease in Childhood 96, 1008–1013. 21288999 10.1136/adc.2010.191882 PMC3195296

[mcn12124-bib-0018] Lima M.A. , Oliveira M.A. & Ferreira Hda S. (2010) [Reliability of anthropometric data obtained in children seen at the Primary Public Healthcare Service Network in Alagoas, Brazil]. Revista Brasileira de Epidemiologia 13, 69–82. 20683556 10.1590/s1415-790x2010000100007

[mcn12124-bib-0019] Ministry of Health (2006) Taking the Kenya Essential Package for Health to the Community: A strategy for the delivery of level one services. Nairobi, Kenya.

[mcn12124-bib-0020] Morris S.S. & Flores R. (2002) School height censuses are reliable and valid tools for small‐area targeting of nutrition interventions in Honduras. The Journal of Nutrition 132, 1188–1193. 12042432 10.1093/jn/132.6.1188

[mcn12124-bib-0021] Mwangome M.K. , Fegan G. , Mbunya R. , Prentice A.M. & Berkley J.A. (2012) Reliability and accuracy of anthropometry performed by community health workers among infants under 6 months in rural Kenya. Tropical Medicine and International Health 17, 622–629. 22364555 10.1111/j.1365-3156.2012.02959.x PMC3963456

[mcn12124-bib-0022] Myatt M. , Khara T. & Collins S. (2006) A review of methods to detect cases of severely malnourished children in the community for their admission into community‐based therapeutic care programs. Food and Nutrition Bulletin 27, S7–S23. 17076211 10.1177/15648265060273S302

[mcn12124-bib-0023] Ngirabega J.D. , Hakizimana C. , Wendy L. , Munyanshongore C. , Donnen P. & Dramaix‐Wilmet M. (2010) [Reliability of anthropometric measurements performed by community nutrition workers in a community‐based pediatric growth‐monitoring program in rural Rwanda]. Revue D'epidemiologie et de Sante Publique 58, 409–414. 10.1016/j.respe.2010.07.002 21094002

[mcn12124-bib-0024] Oza‐Frank R. , Hade E.M. & Conrey E.J. (2012) Inter‐rater reliability of ohio school‐based overweight and obesity surveillance data. Journal of the Academy of Nutrition and Dietetics 112, 1410–1414. 22939442 10.1016/j.jand.2012.06.006

[mcn12124-bib-0025] Poustie V.J. , Watling R.M. , Ashby D. & Smyth R.L. (2000) Reliability of percentage ideal weight for height. Archives of Disease in Childhood 83, 183–184. 10906035 10.1136/adc.83.2.183 PMC1718419

[mcn12124-bib-0026] Rosenberg S.N. , Verzo B. , Engstrom J.L. , Kavanaugh K. & Meier P.P. (1992) Reliability of length measurements for preterm infants. Neonatal Network 11, 23–27. 1549073

[mcn12124-bib-0027] Shrout P.E. & Fleiss J.L. (1979) Intraclass correlations: uses in assessing rater reliability. Psychological Bulletin 86, 420–428. 18839484 10.1037//0033-2909.86.2.420

[mcn12124-bib-0028] Stomfai S. , Ahrens W. , Bammann K. , Kovacs E. , Marild S. , Michels N. *et al* (2011) Intra‐ and inter‐observer reliability in anthropometric measurements in children. International Journal of Obesity (2005) 35 (Suppl. 1), S45–S51. 10.1038/ijo.2011.34 21483422

[mcn12124-bib-0029] Vegelin A.L. , Brukx L.J. , Waelkens J.J. & Van Den Broeck J. (2003) Influence of knowledge, training and experience of observers on the reliability of anthropometric measurements in children. Annals of Human Biology 30, 65–79. 12519655 10.1080/03014460210162019

[mcn12124-bib-0030] Velzeboer M.I. (1979) *The use of arm circumference for simplified screening for malnutrition by minimally trained health workers* . Dr.P.H., University of Texas. 10.1093/tropej/29.3.159 6876236

[mcn12124-bib-0031] Velzeboer M.I. , Selwyn B.J. , Sargent F. 2nd , Pollitt E. & Delgado H. (1983) The use of arm circumference in simplified screening for acute malnutrition by minimally trained health workers. Journal of Tropical Pediatrics 29, 159–166. 6876236 10.1093/tropej/29.3.159

[mcn12124-bib-0032] Voss L.D. & Bailey B.J. (1994) Equipping the community to measure children's height: the reliability of portable instruments. Archives of Disease in Childhood 70, 469–471. 8048813 10.1136/adc.70.6.469 PMC1029862

[mcn12124-bib-0033] Voss L.D. , Wilkin T.J. , Bailey B.J. & Betts P.R. (1991) The reliability of height and height velocity in the assessment of growth (the Wessex Growth Study). Archives of Disease in Childhood 66, 833–837. 1863094 10.1136/adc.66.7.833 PMC1793248

[mcn12124-bib-0034] Walker C.L. , Rudan I. , Liu L. , Nair H. , Theodoratou E. , Bhutta Z.A. *et al* (2013) Global burden of childhood pneumonia and diarrhoea. Lancet 381, 1405–1416. 23582727 10.1016/S0140-6736(13)60222-6 PMC7159282

[mcn12124-bib-0035] Wang J. , Thornton J.C. , Bari S. , Williamson B. , Gallagher D. , Heymsfield S.B. *et al* (2003) Comparisons of waist circumferences measured at 4 sites. The American Journal of Clinical Nutrition 77, 379–384. 12540397 10.1093/ajcn/77.2.379

[mcn12124-bib-0036] Warner J.T. (2000) Reliability of indices of weight and height in assessment of nutritional state in children. Lancet 356, 1703–1704. 11095255 10.1016/S0140-6736(00)03201-3

[mcn12124-bib-0037] West J. , Manchester B. , Wright J. , Lawlor D.A. & Waiblinger D. (2011) Reliability of routine clinical measurements of neonatal circumferences and research measurements of neonatal skinfold thicknesses: findings from the Born in Bradford study. Paediatric and Perinatal Epidemiology 25, 164–171. 21281329 10.1111/j.1365-3016.2010.01181.x PMC3532621

[mcn12124-bib-0038] WHO (2006) Reliability of anthropometric measurements in the WHO Multicentre Growth Reference Study. Acta Paediatrica (Oslo, Norway: 1992). Supplement 450, 38–46. 10.1111/j.1651-2227.2006.tb02374.x 16817677

[mcn12124-bib-0039] WHO & UNICEF (2009) *WHO child growth standards and the identification of severe acute malnutrition in infants and children* . [Online]. Geneva: World Health Organisation. Available at: http://www.who.int/nutrition/publications/severemalnutrition/9789241598163_eng.pdf (Accessed 23 April 2012).

